# Identification and functional characterization of ABCC transporters for Cd tolerance and accumulation in *Sedum alfredii* Hance

**DOI:** 10.1038/s41598-020-78018-6

**Published:** 2020-12-01

**Authors:** Tongyu Feng, Xuelian He, Renying Zhuo, Guirong Qiao, Xiaojiao Han, Wenmin Qiu, Linfeng Chi, Dayi Zhang, Mingying Liu

**Affiliations:** 1 Research Institute of Subtropical of Forestry, Chinese Academy of Forestry, Hangzhou, 311400 People’s Republic of China; 2 School of Basic Medical Sciences, Zhejiang Chinese Medical University, Hangzhou, 310053 People’s Republic of China; 3 School of Environment, Tsinghua University, Beijing, 100084 People’s Republic of China

**Keywords:** Pollution remediation, Plant stress responses

## Abstract

Cd is one of the potential toxic elements (PTEs) exerting great threats on the environment and living organisms and arising extensive attentions worldwide. *Sedum alfredii* Hance, a Cd hyperaccumulator, is of great importance in studying the mechanisms of Cd hyperaccumulation and has potentials for phytoremediation. ATP-binding cassette sub-family C (ABCC) belongs to the ABC transporter family, which is deemed to closely associate with multiple physiological processes including cellular homeostasis, metal detoxification, and transport of metabolites. In the present work, ten ABCC proteins were identified in *S. alfredii* Hance, exhibiting uniform domain structure and divergently clustering with those from *Arabidopsis*. Tissue-specific expression analysis indicated that some *SaABCC* genes had significantly higher expression in roots (*Sa23221* and *Sa88F144*), stems (*Sa13F200* and *Sa14F98*) and leaves (*Sa13F200*). Co-expression network analysis using these five *SaABCC* genes as hub genes produced two clades harboring different edge genes. Transcriptional expression profiles responsive to Cd illustrated a dramatic elevation of *Sa14F190* and *Sa18F186* genes. Heterologous expression in a Cd-sensitive yeast cell line, we confirmed the functions of *Sa14F190* gene encoding ABCC in Cd accumulation. Our study performed a comprehensive analysis of ABCCs in *S. alfredii* Hance, firstly mapped their tissue-specific expression patterns responsive to Cd stress, and characterized the roles of *Sa14F190* genes in Cd accumulation.

## Introduction

Soil contamination by potential toxic elements (PTEs) is a representative of human-induced disturbance on natural biogeochemical cycles and a worldwide threat to all living beings^1–4^. Among all PTEs, Cd is deemed to be more threatening for the resemblance in the valence state of nutrient ions^5^. Cd could exert detrimental effects on microbes and plant morphological, metabolic and physiological procedures by interfering the normal molecular and cellular processes, such as interacting with vital biochemical molecules and generating excessive reactive oxygen species (ROS)^6,7^. Animals and humans are exposed to such risks through the food chain^8,9^. Therefore, decontaminating Cd is of great urgency to minimize its harmful impacts but is a challenge regarding the cost and technical bias^10,11^. Approaches that can effectively remediate Cd-contaminated soils are urgently required.

So far, some physical and chemical approaches have been employed to reclaim Cd-contaminated sites, such as soil washing^12^ and chemical immobilization^13^. However, these methods suffer from several limitations, *e.g.*, high cost, low efficiency, poor long-term stability, and loss of soil functions owing to the change in soil physicochemical and biological properties^14,15^. Biological strategies utilizing plants to extract, immobilize and eliminate PTEs from soils have been developed in recent years, including phytoremediation using native hyperaccumulators^16–18^. For instance, soil inoculation of the Cd-hyperaccumulator *Sedum alfredii* with dichlorodiphenyltrichloroethane-degrading microbes decreased the concentrations of Cd from 0.695 to 0.479 mg/kg over an 18-month period^19^. A two-year phytoremediation project around Huanjiang River using As and Pb hyperaccumulator *Pteris vittata* and Cd hyperaccumulator *S. alfredii* Hance achieved satisfactory PTEs removal efficiency that available As, Cd and Pb was reduced by 55.3%, 85.8% and 30.4%, respectively^20^.

Although more than 700 hyperaccumulators have been identified^21^, there are only rare Cd hyperaccumulators, *e.g.*, *Noccaea caerulescens* and *Arabidopsis halleri*^22^, *Solanum nigrum* L^23^, *Viola baoshanensis*^24^, *Sedum plumbizincicola*^25^, *S. alfredii* Hance^26^ and newly discovered *Lantana camara* L^27^. Among them, *S. alfredii* Hance is a Zn/Cd co-hyperaccumulator native to China, exhibiting remarkable traits of accumulating up to 6,500 mg/kg (dry weight, DW) of Cd and 29,000 mg/kg (DW) of Zn in its stems without displaying significant toxicity symptoms, and the maximum Cd concentration in leaves can reach 9,000 mg/kg (DW)^28,29^. Numerous studies have attempted to elucidate the uptake, translocation, localization and detoxification of Cd in *S. alfredii* Hance^30–33^, and the transporters involved in these procedures include *SaHMA3* (heavy metal ATPase, HMA)^34^, *SaNRAMP3* (natural resistance-associated macrophage protein, NRAMP)^35^, *SaNRAMP6*^36^, *SaMTP1* (metal tolerance protein, MTP)^37^, *SaZIP4*^38^ and *SaCAX2* (cation exchangers, CAX)^39^.

ATP-binding cassette (ABC) proteins are widely distributed in all phyla and have been comprehensively analyzed in numerous species including human^40^, arthropods^41^, lamprey^42^, monogonont rotifer^43^, *Tetrahymena thermophile*^44^, rice^45,46^, *Arabidopsis*^47^, and *Magnaporthe oryzae*^48^. Most ABC proteins have two-fold-symmetric structures, a hydrophobic trans-membrane domain (TMD) and a cytosolic domain containing a nucleotide-binding domain (NBD)^49,50^. ABC transporters are multi-functioned with functional conservation existing in members of different classes^51^. Particularly, the C-subfamily of ABC transporters (ABCC) is extensively studied. In human, ABCC proteins are associated with chemical detoxification, disposition, and normal cell physiology^52,53^. In *Saccharomyces cerevisiae*, the ABCC member ScYCF1 is capable of conferring Cd resistance^54^. In plants, ABCCs are reported to associate with detoxification and PTEs sequestration^55–57^, chlorophyll catabolite transport^58^ and ion channel regulation^59^. For example, in *Arabidopsis*, AtABCC1 is responsible for the removal of glutathione-S (GS) conjugates from the cytosol^60,61^. Additionally, *AtABCC1* and *AtABCC2* genes were reported to link with the vascular sequestration of phytochelatin (PC)-Cd(II) and PC-Hg(II)^55^, whereas *AtABCC3* gene encoded a transporter of PC-Cd complexes^57^. A wheat ABCC protein, TaABCC13, plays critical roles in glutathione-mediated detoxification pathway^62^, and OsABCC1 in rice reduces the amount of As in grains by sequestering As in the vacuoles of the phloem companion cells of diffuse vascular bundles^63^. These findings indicate that ABCC members are vital in elucidating PTEs transport and detoxification.

However, studies on ABCC transporters in *S. alfredii* Hance are still lacking, and the members and functions of ABCC family members in *S. alfredii* Hance remain unclear. Hence, in this study, we mapped the putative ABCC genes at genome-scale based on a previously published transcriptomic database^64^ and comprehensively analyzed their homology through sequence phylogeny and protein/motif structure. Tissue-specific expression profiles of ABCC genes responsive to Cd stress and co-expression network analysis were carried out to link their potential roles to Cd tolerance or accumulation. Additionally, the roles of *Sa14F190* gene in Cd tolerance and accumulation were assessed by heterologous expression in yeasts. Our work would enrich the studies of ABCC transporters in *S. alfredii* Hance, providing new clues for its Cd/Zn hyperaccumulating mechanisms.

## Materials and methods

### Identification of ABCC genes in *S. alfredii* Hance

Putative ABCC members in *S. alfredii* Hance were characterized through a Basic Local Alignment Search Tool (BLAST) search against *S. alfredii* Hance annotated transcriptome database consisting of approximately 8.8 Gb sequencing data assembled into 87,721 unigenes with an average length of 586 bp (NCBI accession number, SRP058333)^64^. The transcriptome library was generated from mixed RNA samples of roots, stems and leaves dissected from *S. alfredii* plants grown under control conditions (25–28 °C, 16 h photoperiod) treated with 400 μM CdCl_2_^64^. The amino-acid sequences of *Arabidopsis* AtABCCs were chosen as the queries and further applied in a local BLAST using the Blast + software supplied by National Center for Biotechnology Information (NCBI)^65^. The expressed sequence tag (EST) hits with E-value less than 10^−6^ were considered as significant ones and their sequences were screened for representative domain signature (Walker A, Walker B, and ABCC-MRP like ATPase domains) as defined in the NCBI conserved domain database (CDD). The location of NBD and TMD was scanned against a large collection of protein families, each represented by multiple sequence alignments and hidden Markov models (PFAM) database version 28.0 using biosequence analysis using profile hidden Markov models (HMMER) v3.1^66^, and only those with confirmed structure features of ABCCs were applied to further analysis. The chosen EST hits were further filtered by removing redundant sequences and annotated with functions using a simple modular architecture research tool (SMART, http://smart.embl-heidelberg.de)^67^ and PFAM (http://pfam.xfam.org/)^68^.

### Phylogenetic analysis, protein structure and conserved motif analysis of SaABCCs

To verify the evolutionary location of putative SaABCC members, a phylogenetic tree was constructed using MEGA 7.0^69^ via the neighbor-joining (NJ) method using ABC proteins of *Arabidopsis* as reference^45^. Phylogenetic analysis was carried out to characterize the evolutionary relationships among AtABCCs and SaABCCs with MEGA 7.0.

For the analysis of protein structure, all the identified SaABCCs were firstly searched against the PFAM database (version 28.0)^68^ and further validated by CDD analysis (http://www.ncbi.nlm.nih.gov/Structure/cdd/wrpsb.cgi). The domain composition of each SaABCC protein was subsequently visualized by the software package of Illustrator of Biological Sequences (IBS)^70^. Analysis of conserved motif distributions was performed using the motif analysis online program, Multiple Expectation Maximization for Motif Elicitation (MEME) (http://meme-suite.org/tools/meme)^71^. Besides default parameters, other parameters were set as follows: any number of repetitions, maximum number of motifs = 20, motif wide between 10 and 30. Finally, the protein structures and the distributions of motifs were combined with the phylogenetic tree using the iTOL tool (http://itol.embl.de)^72^. The molecular weight (kDa) and isoelectric point (pI) of each SaABCCs were calculated by the ExPASy program (http://web.expasy.org/compute_pi) and DNAMAN software.

### Quantifications of SaABCCs expression profiles in tissues and response to Cd stress

The hyperaccumulator *S. alfredii* Hance naturally inhabited on an old Pb/Zn mine in Quzhou City (Zhejiang Province, China) were collected and cultured in a greenhouse with a 16 h light/8 h dark cycle at an average temperature of 25–28 °C. To ensure homogeneity, seedlings of *S. alfredii* Hance were asexual propagated and grew in buckets filled with half-strength Hoagland-Arnon solution (pH = 6.0) in an artificial climate growth chamber for 1 months. The stock solution of CdCl_2_ (0.1 M) was prepared by dissolving 22.835 g of CdCl_2_^.^2.5H_2_O in 1.0 L of half-strength Hoagland-Arnon solution and the working concentration of CdCl_2_ (400 μM) was set based on a previous study verifying the reliable internal genes^73^. Subsequently, 48 vigorous and uniform seedlings were randomly divided according to Cd stress. The exposure duration was 0, 0.5, 2, 4, 8, 16, 32 and 48 h, and six individuals were collected for each treatment and washed thoroughly using RNAase-free water. To avoid the influence of rhythm on gene expression, the sampling time was fixed at 14:00 pm and the exposure starting points for each treatment varied according to the exposure duration. Three tissues (whole roots, the middle part of stems, and young leaves) were collected separately and promptly frozen in liquid nitrogen for RNA extraction.

Total RNA was extracted using Total RNA Purification Kit (Norgan Biotek Corp., Ontario, Canada) from roots, stems and leaves of *S. alfredii* Hance, and subsequently treated with RNase-free DNase I (NEB BioLabs, Ipswich, MA, USA) to remove genomic DNA. Then, 3 μg of RNA were used to synthesize the first strand of cDNA using the SuperScript III First-Strand Synthesis System (Invitrogen, Carlsbad, CA, USA) with oligo d(T) primers (Invitrogen, Carlsbad, CA, USA). Quantitative real-time polymerase chain reaction (qRT-PCR) was carried out in triplicates on a 7300 Real-Time PCR System (Applied Biosystems, CA, USA) using SYBR Premix Ex Taq™ (TaKaRa, Dalian, China) in a 20 μL reaction system (2 μL of cDNA reaction mixture, 10 μL of SYBR Premix Ex Taq™, 0.4 μL of ROX Reference Dye, and 0.4 μL of each primer). The amplification conditions were set as follows: denaturation at 94℃ for 10 s, 40 cycles of amplifications (94℃ for 5 s, 60℃ for 31 s), and a final gradient heating from 60℃ to 95℃ for the melting dissociation curve. Gene encoding ubiquitin conjugating enzyme 9 (UBC9) was selected as the reference gene^73^ and the expression levels of all target genes were adjusted by that of UBC9. The primers are listed in Table S2 (Supporting Information).

To uncover tissue-expression profiles of ten *SaABCC* genes in roots, stems and leaves of *S. alfredii* Hance, the relative expression level was calculated as the ratio of expression level of each gene to the one with the lowest expression level (*Sa14F190* in roots and stems, *Sa88F144* in leaves) as reference. For Cd-responsive expression profiles of *SaABCC* genes in different tissues of *S. alfredii* Hance, the relative abundance of each gene was calculated as the ratio of its expression level under Cd pressure to that without Cd exposure (time = 0). Analysis of variance (ANOVA) was performed using R software (v3.0.3) to test the significance of gene expression comparing to control at *p* = 0.05 level. Significantly up-regulated or down-regulated genes were those with relative abundance > 1.5 or < 0.7 and *p* < 0.05.

### Co-expression network construction

The previously reported transcriptome dataset was constructed from mixed RNA samples of three tissues (roots, stems and leaves) from Cd-stressed (400 μM) *S. alfredii* plants^64^. Genes responding to Cd stress were annotated and a co-expression network was constructed to identify the modules of highly correlated genes responding to Cd stress using weighted gene co-expression network analysis (WGCNA) package^64^. Among all hub genes referring to co-expressed ones with strong interconnections, *SaABCC*s were screened and analyzed for their correlated edge genes among the annotated differentially expressed genes. The threshold of Pearson correlation coefficient of FPKM (fragments per kilobase of exon per million reads mapped) values for each gene pair was set at 0.40 and all the edges meeting the criterion were categorized by their GO annotations. The correlations of the identified hub *SaABCC*s and edge genes were visualized using Cytoscape v3.6.1 with the NetworkAnalyzer plugin^74^.

### Yeast-expressing vector construction and heterologous expression of Sa14F190 in yeast

As *Sa14F190* gene dramatically responded to Cd stress and behaved as a hub gene in the co-expression network, it was chosen as the target *SaABCC* genes to characterize the functions in Cd hypertolerance and hyperaccumulation. The open reading frame (ORF) of *Sa14F190* gene was amplified using High Fidelity KOD-Plus DNA Polymerase (Toyobo, Japan) from the cDNA of *S. alfredii* Hance with specific primers listed in Table S2 (Supporting Information). The forward primer *Sa14F190-F* and reverse primer *Sa14F190-R* was supplemented with SpeI and SmaI site, respectively. The PCR products were then gel-purified using a DNA gel extraction kit (Axygen, USA) and digested with SpeI and SmaI. The gel-purified digests were ligated into the yeast expression vector pDR196 previously cut with the same restriction enzymes at 4 °C overnight in a 10 μL reaction system (6 μL of digested PCR products, 2 μL of digested pDR196, 1 μL of 10 × T4 DNAase ligase buffer, and 1 μL of T4 DNAase ligase). The ligase was transformed into Top10 competent cells (TIANGEN, China) and cultured overnight in Luria–Bertani (LB) broth containing ampicillin (100 µg/mL). The positive colonies were further cultured in 1 mL of liquid LB broth supplemented with ampicillin (100 µg/mL) and validated by PCR using the primer pair of pDR196-F (ATGTCCTATCATTATCGTCTA) and pDR196-R (CTTTTCGATCTTTTCGTA). They were further sequenced and the verified plasmids were designated as pDR196-*Sa14F190* which were then transferred into a Cd-sensitive mutant *Saccharomyces cerevisiae* strain BY4742 *Δycf1* (MATα; his3Δ1; leu2Δ0; met15Δ0; ura3Δ0; YDR135c::kanMX4)^54^ by the lithium acetate method^75^. Positive yeast clones surviving on half-strength synthetic dextrose agar plates lacking uracil (SD-U) were further validated by PCR using the primer set of pDR196-F and pDR196-R to confirm the successful transformation of pDR196-*Sa14F190* and designated as *Δycf1*_*Sa14F190*. *Δycf1* containing empty vector was constructed following the same method and named as *Δycf1_EV.*

### Functions of Sa14F190 gene in Cd tolerance and accumulation

The functions of *Sa14F190* gene in Cd tolerance and accumulation were assessed by yeast spotting assay, growth curve and cellular Cd content. In yeast spotting assay, *Δycf1*_*Sa14F190* and *Δycf1_EV* were cultivated in liquid SD-U medium until the optical density at 600 nm (OD_600_) reached 1.0. Cells were then serially diluted to OD_600_ of 0.1, 0.01, 0.001, 0.0001 and 0.00001, spotted on SD-U agar plates containing 0 and 30 µM CdCl_2_ respectively, and cultured at 28 °C for 3 days before photographs were taken. The inhibition effects of Cd on cell growth was measured in liquid SD-U medium containing 10 µM CdCl_2_, and both *Δycf1*_*Sa14F190* and *Δycf1_EV* were cultured at 28 °C for 72 h. OD_600_ was measured every 12 h.

To compare cellular Cd contents in *Δycf1*_*Sa14F190* and *Δycf1_EV*, they were cultured in liquid SD-U medium with 10 µM CdCl_2_ for 48 h, harvested by centrifugation at 5 000 × g for 10 min and washed three times with distilled water. The cell pellets were then washed by Na_2_EDTA (0.1 mM) for more than three times to remove residual Cd on cell surface. The washed cells were then dried at 65 °C until the weight was constant and finally digested by HNO_3_ and perchloric acid (9:1, v:v) to determine Cd content using inductively coupled plasma mass spectrometry (ICP-MS, 7500a, Agilent, Santa Clara, CA, USA). Cd content was expressed in terms of mg/g (dry weight, DW).

## Results

### Identification and classification of SaABCC genes in *S. alfredii* Hance

**Figure 1:**
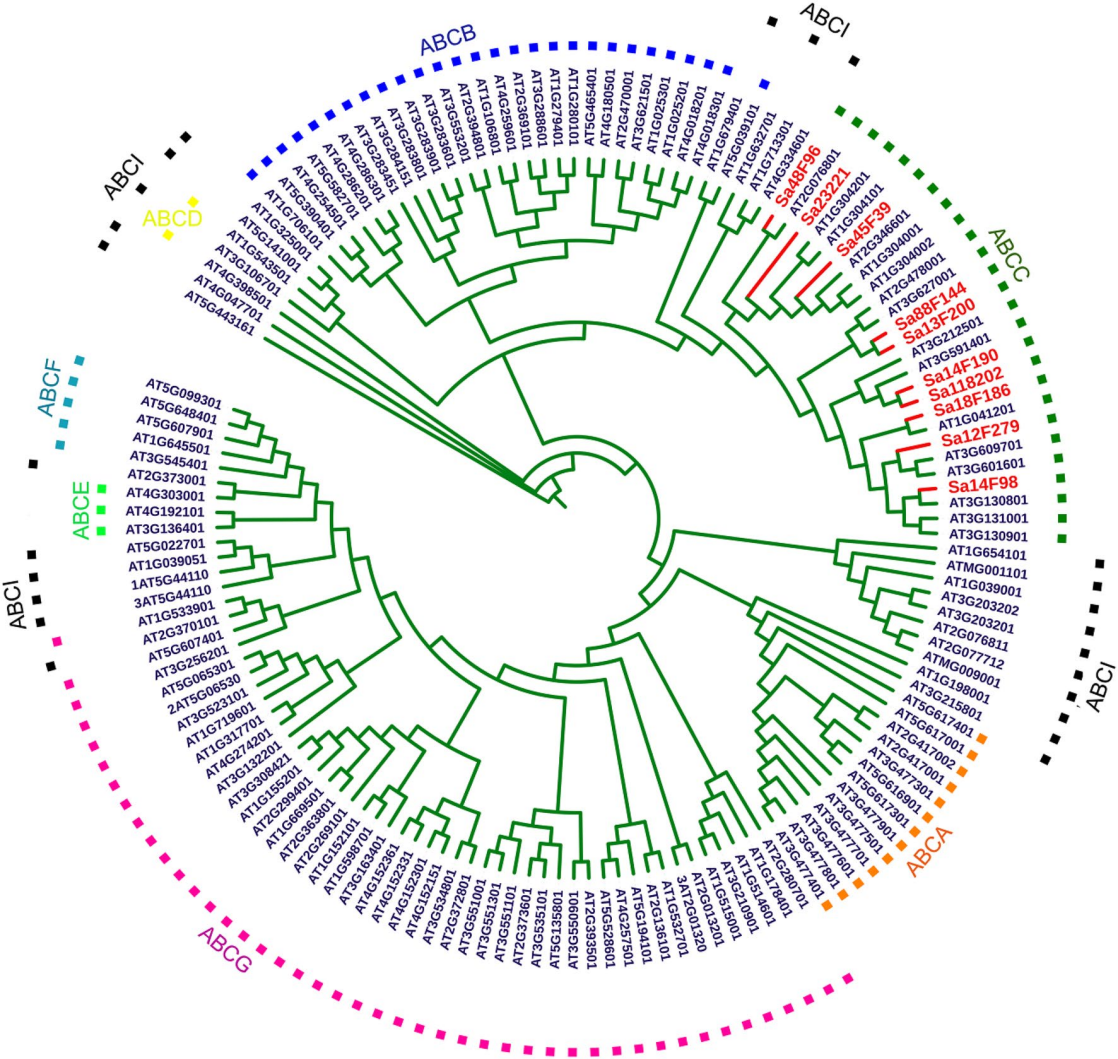
Phylogenetic tree for ABCCs genes identified in *S. alfredii* Hance (red) using ABC members of *Arabidopsis* (blue) as reference. The phylogenetic tree is constructed using MEGA 7.0 via the neighbor-joining (NJ) method using ABC proteins of *Arabidopsis* as reference.

In total, ten genes belonging to ABCC subfamily were identified from the transcriptome database of *S. alfredii* Hance after removing those partial or redundant sequences, and the detailed sequence information is deposited in Genbank (accession numbers listed in Table S2, Supporting Information). These *SaABCC* genes were named after their original IDs, and a phylogenetic tree was constructed using *SaABCC* genes and all ABC family members of *Arabidopsis* (Fig. 1). The results illustrate that members of *AtABC* genes are segregated into 11 clusters and *SaABCC* genes cluster with the C-subfamily of *AtABCs*.

### Protein domains and motif analysis of SaABCCs

**Figure 2:**
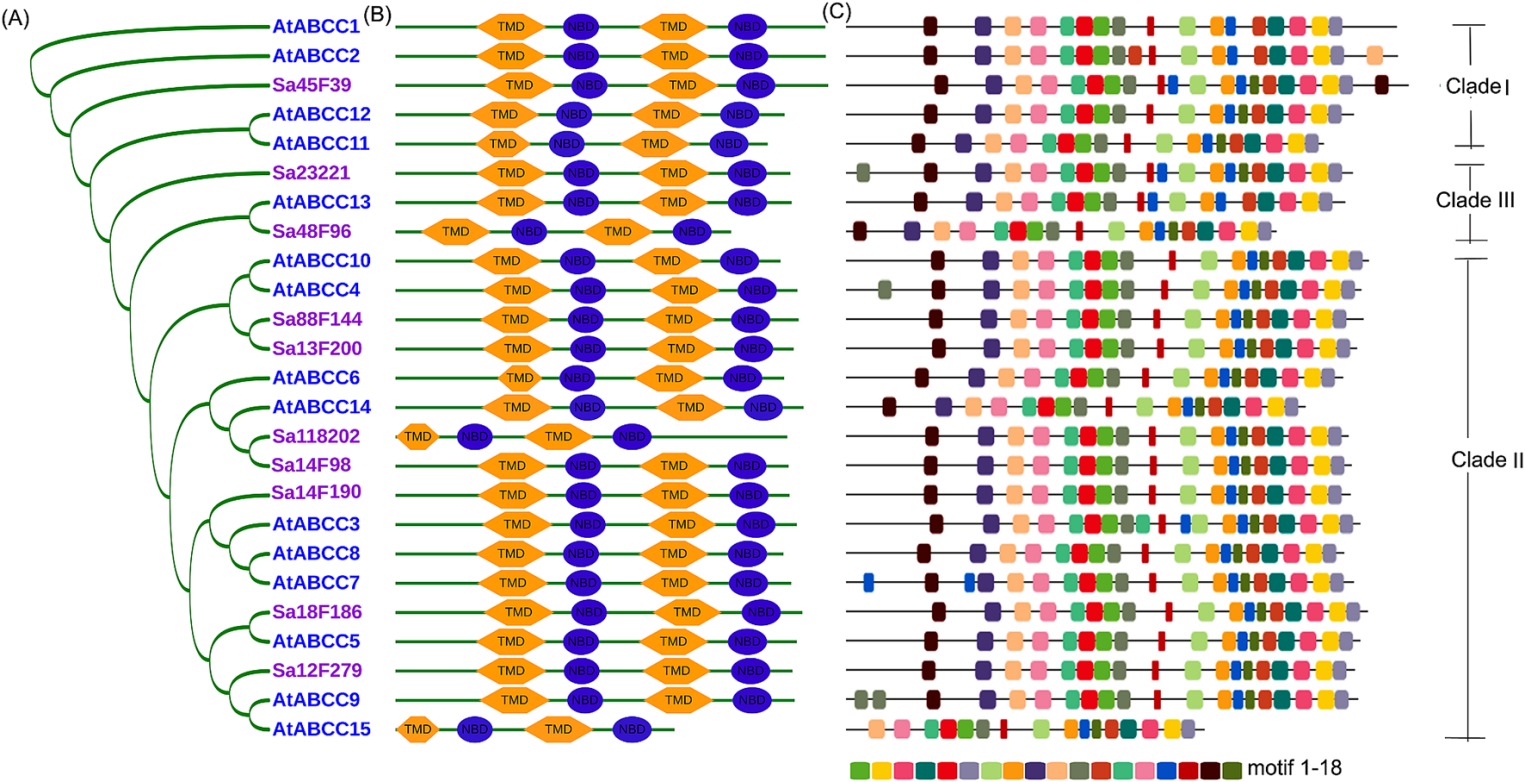
Phylogenetic analysis of ten identified SaABCC proteins from *S. alfredii* Hance and fifteen AtABCCs from *Arabidopsis* using the neighbor-joining (NJ) method. (**A**) Neighbor-joining tree. (**B**) Structures of ABCC proteins within each subfamily. (**C**) MEME motif distribution of each protein. The phylogenetic tree is constructed using MEGA 7.0 via the neighbor-joining (NJ) method and the location of NBD and TMD is scanned against a large collection of protein families by multiple sequence alignments and hidden Markov models (PFAM) database version 28.0 using biosequence analysis using profile hidden Markov models (HMMER) v3.1.

The predicted amino acid lengths of SaABCCs range from 1,266 to 1,655 aa (Table S2, Supporting Information), sharing 29.4% to 83.6% similarity with those of AtABCCs. Phylogenetic analysis visualized the relationships between SaABCCs and members of AtABCCs, as illustrated in Fig. 2A. According to the three clades (I, II and III) of AtABCCs^76^, different portions of members are assigned between AtABCCs (4/15 for clade I, 10/15 for clade II, 1/15 for clade III) and SaABCCs (1/10 for clade I, 7/10 for clade II, 2/10 for clade III). Sa45F39 is clustered in clade I including AtABCC1, AtABCC2, AtABCC11 and AtABCC12, whereas Sa48F96 and Sa23221 are clustered in clade III with AtABCC13 (Fig. 2A). All the other SaABCCs belong to clade II. Sa88F144 and Sa13F200 are clustered together, showing a close relationship with AtABCC4 and AtABCC10, whereas Sa14F98 and Sa118202 group together with AtABCC14 and AtABCC6 (Fig. 2A). Sa14F190 is located near the subclade of AtABCC3, AtABCC7 and AtABCC8, and Sa12F279 is located close to AtABCC9 and AtABCC15 (Fig. 2A). The analysis of functional domains reveals the presence of TMD and NBD in a topological pattern of TMD-NBD-TMD-NBD (Fig. 2B). The three signature motifs (Walker A, Walker B and ABC transporter consensus motif) are also found in NBD domains of all SaABCCs. MEME scan suggests 16–18 motifs in SaABCCs and AtABCCs (Fig. 2C), and their number and distribution all follow the order of 17-9-10-14-13-5-1-11-16-7-8-15-18-12-4-3-2-6 among all SaABCCs, except for Sa23221 and Sa45F39 (Fig. 2C).

### Tissue expression of SaABCCs

**Figure 3:**
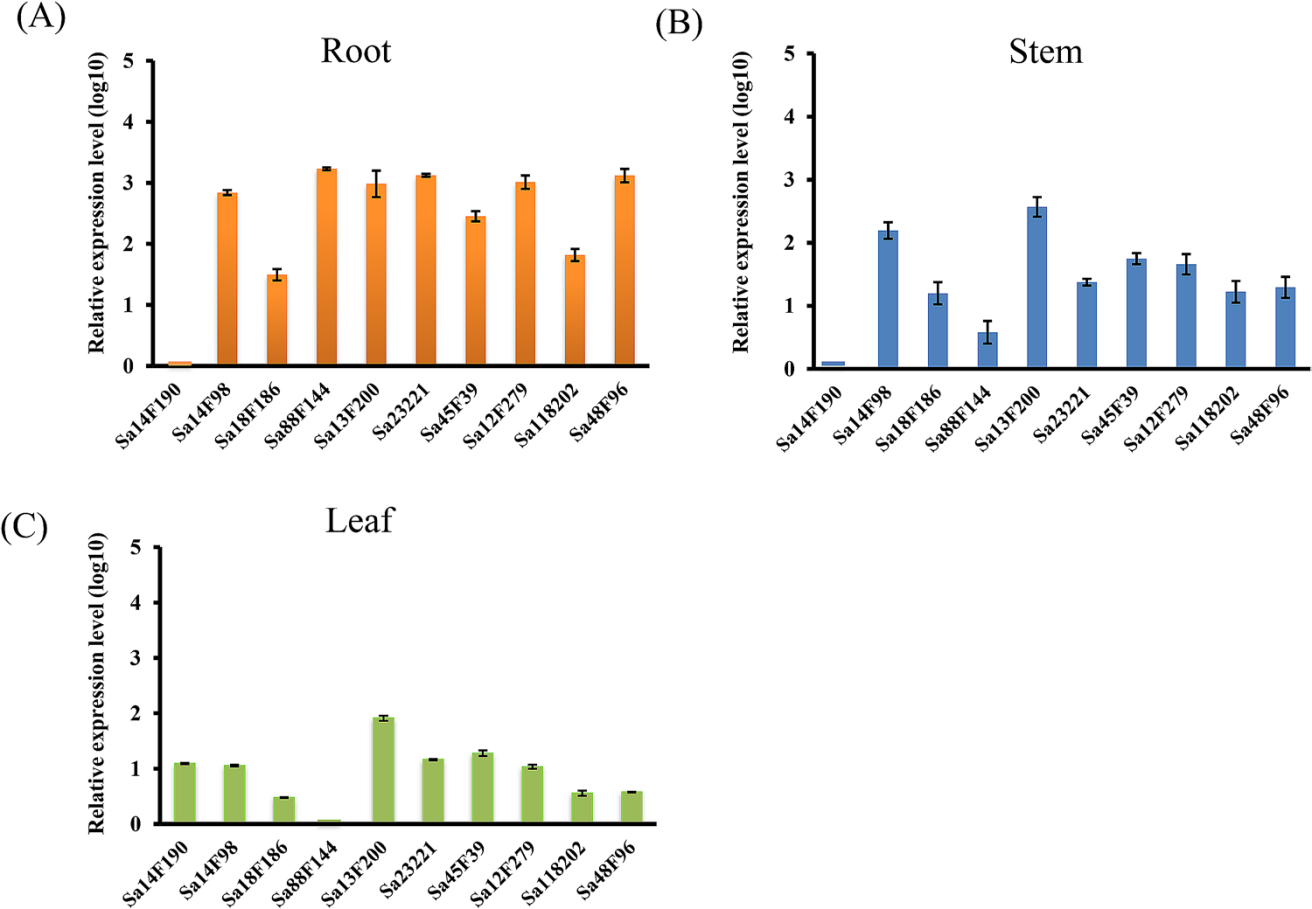
Tissue-expression profiles of ten *SaABCC* genes in roots (**A**), stems (**B**) and leaves (**C**) of *S. alfredii* Hance. The expression levels of all target genes were adjusted by that of UBC9.

The identified *SaABCC* genes exhibited a diverse tissue-expression profile, as illustrated in Fig. 3. Five genes showing root-specific expression included *Sa88F144*, *Sa23221*, *Sa48F96*, *Sa14F98* and *Sa12F279*. Only one gene (*Sa14F190*) had a leaf-specific expression pattern, and no gene exhibited specific expression in stems. The other four genes showed no obvious tissue prevalence although they differed in the expression levels. For instance, *Sa13F200* and *Sa18F186* were highly and lowly expressed in all three tissues.

In roots, the expression levels of *SaABCCs* had more variation than those in stems and leaves. Comparing the expression level of *Sa14F190* gene in roots which was the lowest among all genes, the relative expression level of *Sa23221* and *Sa88F144* was over 2,000 times higher (*p* < 0.05), followed by *Sa48F96*, *Sa12F279*, *Sa13F200* and *Sa14F98* genes which exhibited 500 times stronger expression (*p* < 0.05, Fig. 3A). In stems, *Sa13F200* and *Sa14F98* genes had the strongest expression, over 200-fold higher than that of *Sa14F190* gene (*p* < 0.05, Fig. 3B). The expression levels of *SaABCCs* in leaves fell in a relatively narrow range and the highest expressed one was *Sa13F200* (Fig. 3C).

### Cd-stressed profiles under different treatment time

**Figure 4:**
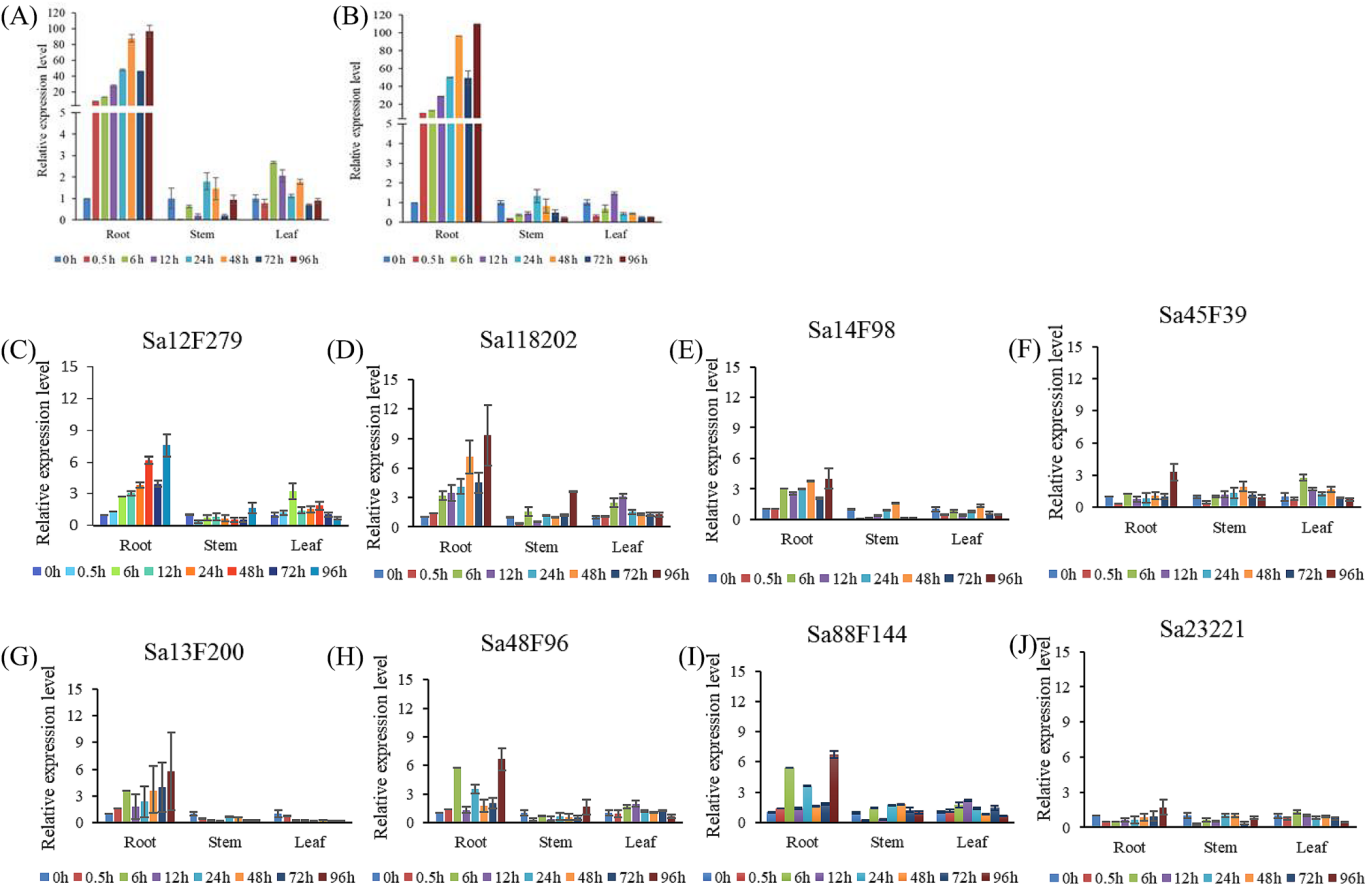
Cd-responsive expression profiles of *SaABCC* genes in different tissues of *S. alfredii* Hance. (**A**) *Sa14F190*, (**B**) *Sa18F186*, (**C**) *Sa12F279*, (**D**) *Sa118202*, (**E**) *Sa14F98*, (**F**) *Sa45F39*, (**G**) *Sa13F200*, (**H**) *Sa48F96*, (**I**) *Sa88F144*, (**J**) *Sa23221*. The relative abundance of each gene was calculated as the ratio of its expression level under Cd pressure to that without Cd exposure (time = 0). The expression levels of all target genes were adjusted by that of UBC9. Significantly up-regulated or down-regulated genes were those with relative abundance > 1.5 or < 0.7 and *p* < 0.05 (ANOVA).

The expression profiles postexposure to Cd showed different responses of these *SaABCC* genes to Cd stress. More precisely, nine *SaABCC* genes (*Sa14F190*, *Sa18F186*, *Sa12F279*, *Sa14F98*, *Sa118202*, *Sa13F200*, *Sa48F96*, *Sa88F144* and *Sa45F39*) only exhibited upregulated patterns in the presence of Cd in roots (*p* < 0.05), but not in stems and leaves (*p* > 0.05). Particularly, *Sa14F190*, *Sa18F186*, *Sa12F279*, *Sa14F98* and *Sa118202* genes exhibited a continuous up-regulation throughout the whole stress procedures in roots (Fig. 4A,E), and the peak expression of *Sa14F190* and *Sa18F186* genes was over 100-fold induced (Fig. 4A,B). *Sa12F279*, *Sa14F98* and *Sa25F86* genes displayed moderate up-regulation patterns in roots (*p* < 0.05, Fig. 4C,E), and the other four genes (*Sa13F200*, *Sa48F96*, *Sa88F144* and *Sa45F39*) exhibited slight but significant positive response to Cd stress at certain time points (*p* < 0.05, Fig. 4F, I), whereas the levels of *Sa23221* gene did not show significant change (the relative abundance = 1.6, *p* > 0.05, Fig. 4J). All these genes did not show significant up-regulation in stems or leaves postexposure to Cd (*p* > 0.05, Fig. 4).

### Co-expression network analysis

**Figure 5:**
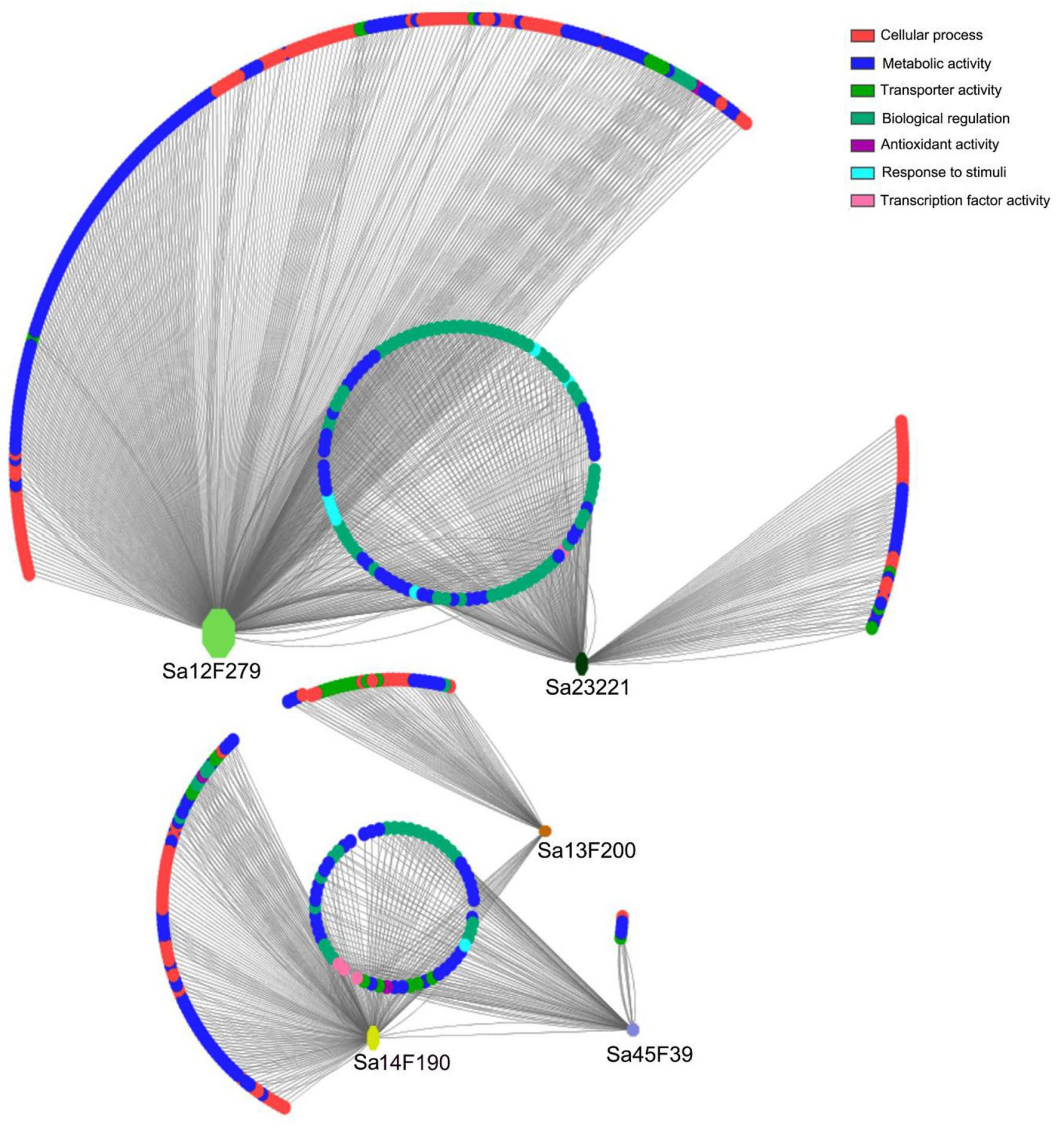
Co-expression network of *SaABCC* genes. Nodes representing individual genes and edges indicate significant co-expressions between genes. Genes involved in the same biological process are grouped together and highlighted with different colors, including cellular process (red), metabolic activity (blue), transporter activity (dark green), biological regulation (light green), antioxidant activity (purple), response to stimulus (baby blue) and transcription factor (purple). The figure is drawn using Cytoscape v3.6.1 with the NetworkAnalyzer plugin.

Among all the identified *SaABCC* genes, *Sa13F200*, *Sa45F39*, *Sa23221*, *Sa14F190* and *Sa12F279* genes are characterized as hub genes showing strong interconnections with those co-expressed genes according to the co-expression network analysis (Table S1, Supporting information). The co-expression network harbors 551 nodes and 1249 connections (Fig. 5), and the edge genes have the functions associated with metabolic process (549 edges), cellular process (420 edges), biological regulation (115 edges), transporter activity (60 edges), response to stimuli (55 edges) and transcription factor (50 edges).

Through the circling layout, the five *SaABCC* hub genes are categorized into two clades. *Sa23221* and *Sa12F279* genes in clade I share with some identical nodes mainly categorized to metabolic process, transporter activity and response to stimuli (Fig. 5). Nodes of clade II (*Sa13F200*, *Sa45F39* and *Sa14F190*) cover the functions linked to metabolic process, transporter activity and transcription factor. *Sa12F279* gene has 567 edges and is the largest module in the co-expression network, including 225 edges involved in metabolic process, 212 edges participating in cellular process, 39 edges associated with biological regulation, 21 edges executing transporter activity, 17 edges taking parts in response to stimuli and 14 edges related to transcription factor. *Sa13F200* gene has more edges with response to stimuli (8/79) and transcription factor (6/79). *Sa14F190* gene is assigned 254 edges mostly related to metabolic process (101 edges) and cellular process (75 edges), and harbors 16 edges executing transporter activity, 13 edges responding to stimuli and 11 edges associated with transcription factor. Most edges of *Sa23221* and *Sa45F39* genes are linked to metabolic process (122/251 and 44/108, respectively) and cellular process (89/251 and 28/108, respectively).

### Heterologous expression and function verification of Sa14F190 gene in Cd-sensitive yeast cells

**Figure 6:**
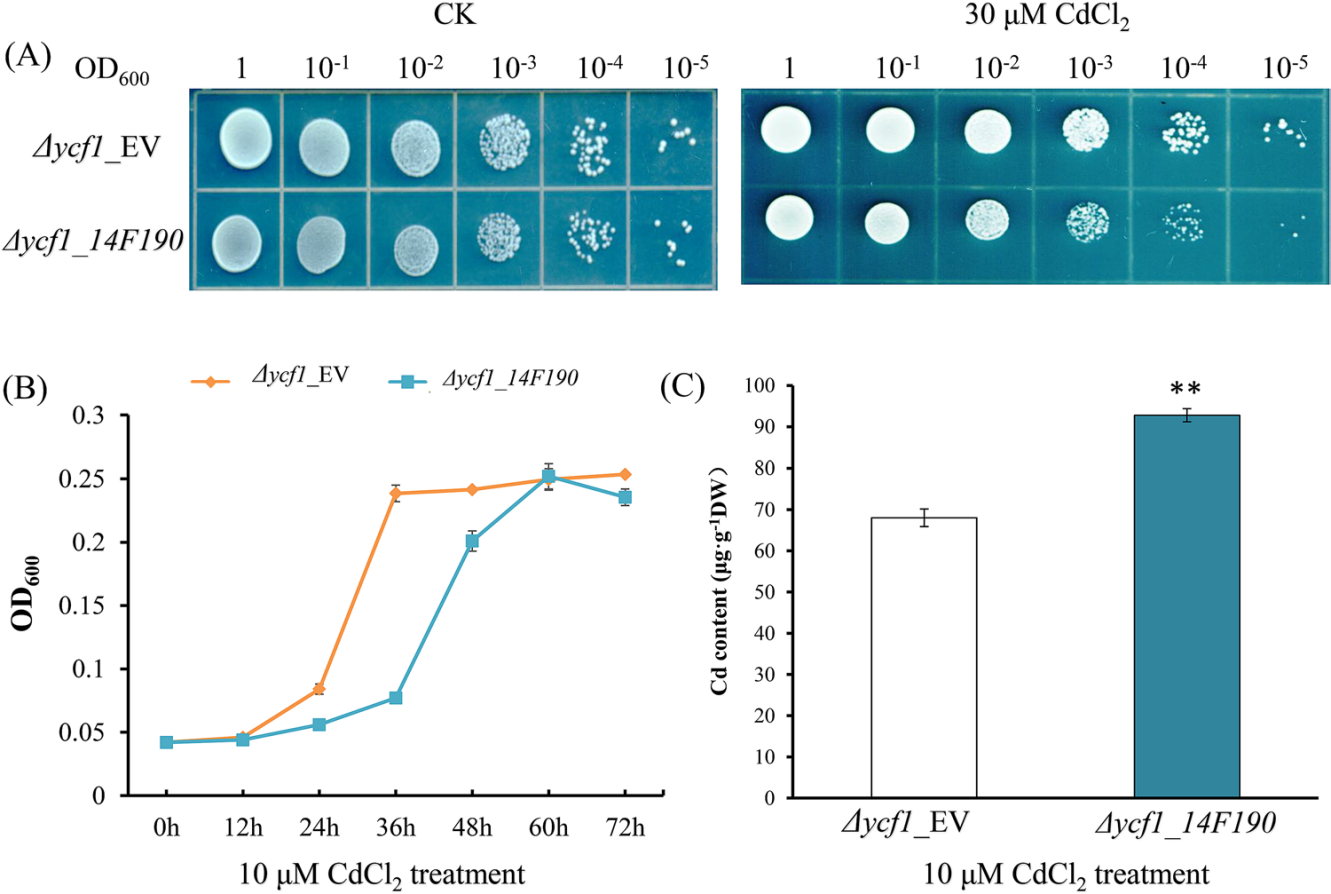
Function validation of *Sa14F190* gene in yeasts. (**A**) *Sa14F19*-conferred Cd sensitivity of *Δycf1*_*Sa14F190* and *Δycf1_EV*. OD_600_ of cell suspensions is 1.0, 0.1, 0.01, 0.001, 0.0001 and 0.00001 from left to right. (**B**) Growth curves of *Δycf1*_*Sa14F190* and *Δycf1_EV* in liquid SD-U supplemented with 10 µM CdCl_2_. Data are means ± standard deviations (SD) from three independent experiments. (**C**) Cd contents in *Δycf1*_*Sa14F190* and *Δycf1_EV* after 48-h growth in liquid SD-U supplemented with 10 µM CdCl_2_. Data are means ± SD from at least three independent biological replicates. Two asterisks indicate a significant difference at p < 0.01 comparing to *∆ycf1_EV*.

As *Sa14F190* gene was strongly induced in roots by Cd stress and characterized as a hub gene with edges related to transporter activity and response to stimuli, it was selected for constructing yeast-expressing cell lines to prove its roles in Cd tolerance and accumulation. Results from both spotting assay and growth curves illustrated that Cd exerted a more profound inhibition effect on *∆ycf1_Sa14F190* than *∆ycf1_EV* (Fig. 6). Both *∆ycf1_Sa14F190* and *∆ycf1_EV* grew vigorously without Cd stress in the spotting assay, whereas the growth of *∆ycf1_Sa14F190* was more significantly retarded under Cd stress than *∆ycf1_EV* (Fig. 6A). Similarly, *∆ycf1_Sa14F190* and *∆ycf1_EV* exhibited different growth curves in liquid medium supplemented with CdCl_2_ (Fig. 6B) that *∆ycf1_Sa14F190* took much longer time to reach the post-exponential phase (60 h) than that of *∆ycf1_EV* (36 h).

To assess whether the growth inhibition was attributing to Cd accumulation, cellular Cd content was compared between *∆ycf1_Sa14F190* and *∆ycf1_EV* (Fig. 6C). Cd content in *∆ycf1_Sa14F190* cells was 92.8 μg/g (DW), significantly higher than that in *∆ycf1_EV* cells (68.5 μg/g DW, p < 0.01). The results suggested that the expression of *Sa14F190* gene may facilitate the import of Cd inside the yeasts.

## Discussion

ABC proteins are powerful transporters driving the exchange of compounds across many different biological membranes and the C-subfamily of ABC proteins is an important component^77^. In human, ABCC transporters have been studied extensively on substrate specificity, tissue expression and transport kinetics to provide insights into cellular functions, drug discovery and development^52,78^. In plants, ABCC transporters are originally defined as vacuolar pumps of GS conjugates, and deemed to associate with detoxification, PTEs sequestration, chlorophyll catabolite transport and ion channel regulation^76,79^.

As a plant hyperaccumulating Cd, *S. alfredii* Hance harbors numerous genes related to metal transport or detoxification and requires intense attentions. Several characterized transporter genes include *SaHMA3*^34^, *SaZIP4*^38^ and *SaNramp6*^36^. Recent reports from *Arabidopsis* confer the roles of ABCC transporters as major detoxifiers sequestering metal-chelators into the plant vacuoles^57,58^, suggesting that ABCC proteins might contribute to PTEs hyperaccumulation and hypertolerance. However, the physiological functions of ABCC proteins are still scarce in metal hyperaccumulators, and it is of great importance to address their roles in *S. alfredii* Hance. In the present study, the composition and diversification of ABCC subfamily in *S. alfredii* Hance were identified using bioinformatics tools and their expression profiles were characterized for their possible roles in Cd tolerance and accumulation.

Among the identified ten proteins belonging to the ABCC subfamily, Sa45F39 is largest (1630 aa) and Sa48F96 is the smallest one (1024 aa). Similar size distribution is observed in *Arabidopsis*^45^, rice^46^ and wheat^62^. However, the number of *ABCC* genes in *S. alfredii* Hance is fewer than those reported species. For instance, there are 15 ABCC proteins in *Arabidopsis*^45^, 17 in rice^45^, 18 in wheat^62^, 26 in *Vitis vinifera*^80^ and 47 in *Brassica napus*^81^. Therefore, we presume that *S. alfredii* Hance may adopt a more economic strategy of assembling multi-functional genes rather than expanding gene family to cope with the external PTEs stress during the adaptive evolution. This phenomenon is also observed for leucine-rich repeat receptor-like protein kinase (LRR-RLK) gene family which is also smaller in *S. alfredii* Hance than other plant species^82^. Nevertheless, these results are obtained from the transcriptomics rather than a complete genome sequencing data, which limits more precise evaluation of gene numbers especially for gene families composed by long proteins such as ABCC subfamily.

The analysis on phylogeny and protein structure provides hints on the possible functions of *SaABCC* genes. Categorized into the division of *AtABCC* clade (Fig. 1), *SaABCC* genes are members of ABCC subfamily. All SaABCCs have similar distribution of TMD and NBD as that of AtABCCs, possessing similar protein length and spacer region (Fig. 2). However, it is worth noticing that members of SaABCCs and AtABCCs fall in different clades (I, II and III)^76^, and SaABCCs are only in clade I and II, suggesting the possibilities of multi-functionality in *S. alfredii* Hance. Additionally, SaABCCs have the identical numbers of MEME motifs and possess uniform distribution except for Sa23221 and Sa45F39 (Fig. 2C), demonstrating a higher conservation than AtABCCs. This may imply that under severe surroundings with PTEs stress, members of ABCC subfamily have prior functions to detoxify PTEs than other roles.

The transcript abundance of *ABCC* genes across tissues helps in understanding their molecular functions. In the present study, six *SaABCC* genes exhibited tissue-specific expression patterns in the absence of Cd, five (*Sa88F144*, *Sa23221*, *Sa48F96*, *Sa12F279* and *Sa14F98)* in roots and one (*Sa14F190*) in leaves (Fig. 3). Among them, *Sa14F98* may participate in the plant growth and development as it is clustered with *AtABCC6* which shows a similar root-specific expression^62^. *Sa14F190* showing the similar expression specificity in leaves as *TaABCC14* and *TaABCC15*^62^ may serve as candidate transporters in leaves. Although the clustering of SaABCCs and AtABCCs hints their functional similarity, there might be functional divergences as well. For example, though *Sa88F144* and *Sa13F200* are clustered together with *AtABCC4*, *Sa13F200* was abundantly expressed in all three tissues and *Sa88F144* exhibited a root-specific expression pattern, neither consistent with the low expression level of *AtABCC4* in all tissues^83^. ABCCs in wheat are reported to show preferential expression in specific tissues, *e.g.*, *TaABCC3* in roots, *TaABCC1* in stems, *TaABCC14* and *TaABCC15* in flag leaves^62^. As *TaABCC3* clustered with *AtABCC6* is a gene highly expressed at the initiation point of secondary roots^84^, it is suggested to play roles in root architecture development^62^. Such different expression patterns imply the possibilities of performing other functions apart from getting involved in the control of stomatal movements as *AtABCC4*^83^.

Cd-responsive expression of *SaABCC* genes hints their roles in Cd hyperaccumulation and hypertolerance in *S. alfredii* Hance. In the presence of Cd, *Sa14F190* and *Sa18F186* genes were mostly induced in roots (Fig. 4). *Sa18F186* is segregated with *AtABCC5* which is involved in K^+^ uptake and salt stress tolerance^85^. Although AtABCC5 is regarded as a central regulator of guard cell ion channel^59^, guard cell signaling and phytate storage^86^, rare studies report the participation of AtABCC5 in Cd response. The functional discrepancy between *Sa18F186* and *AtABCC5* may arise from the different leaf structures that *S. alfredii* has fleshy leaves and *Sa18F186* therefore might enroll to combat Cd stress rather than water loss. *Sa14F190* gene falls into the same clade with *AtABCC3* and *AtABCC7*, and AtABCC3 is important vacuolar transporters conferring Cd tolerance in *Arabidopsis*^57^. In addition, a Cd-responding cluster is found comprising *AtABCC3*, *AtABCC6*, *AtABCC7* and *SAT3* (serine acetyl-transferase gene) on chromosome III^84,87^. Although *Sa45F39* did not respond significantly to Cd stress, it is clustered with *AtABCC1*/*AtABCC2*, which are reported to confer the tolerance to As^56^, Cd and Hg in *Arabidopsis*^55^. *AtABCC2* is particularly involved in vacuolar transport of chlorophyll catabolites^58^ and the uptake of cyanidin 3-*O*-glucoside (C3G)^88^. In rice, *OsABCC1* closely related to *AtABCC1*/*AtABCC2* does not confer Cd tolerance; instead, it is involved in As detoxification and reduces the allocation of As in grains^63^. Therefore, *Sa45F39* is also speculated with roles in PTEs tolerance.

Han et al. proposed a comparative analysis of *S. alfredii* Hance transcriptomic datasets and characterized numerous hub genes strongly associated with Cd stress^64^. However, there are lack of details about *SaABCC* hub genes and their connections with other genes. In the present study, a co-expression network was reconstructed and uncovered five *SaABCC* hub genes (*Sa14F190*, *Sa13F200*, *Sa12F279*, *Sa23221* and *Sa45F39*). The overlapping in the edge genes hints the internal connections among *SaABCC* hub genes. For *Sa14F190* gene, the edge genes are associated with metabolic process, cellular process, biological regulation activity, transporter activity, response to stimuli and transcription factor (Fig. 5). To be more precise, the edge genes performing transporter activity include multidrug and toxic compound extrusion (MATE) efflux family protein, zinc/iron transporter, ABC transporter B family member and sulfate transporter. Those participating in metabolic process and cellular process consist of respiratory burst oxidase homolog protein D, CBL-interacting serine/threonine-protein kinase (CIPK), plant cysteine oxidase, endoplasmic reticulum oxidoreductin-1, phospholipid-transporting ATPase 1, GDSL esterase/lipase, and heavy metal-associated isoprenylated plant protein (HIPP). Representative transcription factors include bZIP and WRKY transcription factors. Among them, MATE efflux family protein harbors multitasking abilities in encompassing regulation of plant development, secondary metabolite transport, xenobiotic detoxification, Al tolerance, and disease resistance^89^. Zinc/iron transporters differs in substrate range and specificity, involved in the transport of Zn, Fe, Mn and Cd^90,91^. Members belonging to HIPP, CIPK and WRKY transcription factor are reported to confer Cd tolerance in yeasts^92^. Therefore, the functions of Sa14F190 are activated or magnified through either direct or indirect interactions with these edge genes.

Taking Cd-responsive profiles and co-expression network together, we chose *Sa14F190* to further assess its roles in Cd tolerance and accumulation by heterologous expression in Cd-sensitive yeasts. Under Cd stress, Cd content in *∆ycf1_Sa14F190* was 35.48% higher than that in *∆ycf1_EV* (Fig. 6), indicating the vital roles of *Sa14F190* gene in Cd accumulation. However, *Sa14F190* increased the sensitivity of transformed yeasts instead of enhancing Cd tolerance, evidenced by the retarded growth which may be due to more Cd accumulation. Similar findings are observed for other transporters in *S. alfredii* Hance which all enhanced the Cd sensitivity and Cd accumulation of transformed yeasts, and the increase of cellular Cd content was 22.2% by *SaNramp6*^36^, 25.2% by *SaHMA3*^34^ and 16.4% by *SaCAX2h*^39^. It has been reported that *AtABCC3* could confer Cd tolerance in yeast cells, but not Cd accumulation^93^, consistent with another study on *AtABCC3* in *Arabidopsis*^57^. These findings suggested that members of ABCC subfamily closely located in the phylogenetic tree might have functional divergence, and Cd tolerance and accumulation are mediated by different pathways.

In summary, the ABCC transporter *Sa14F190* gene is responsible for Cd hyperaccumulation and other *SaABCC*s might have diverse roles in the tolerance or accumulation of PTEs. Members of ABCC transporter are a genetic pool of candidates encompassing strong ability to transport, tolerate or accumulate Cd and other PTEs for phytoremediation. This work applies bioinformatic analysis to provide a preliminary study uncovering the interesting functions of ABCCs members in *S. alfredii* Hance, and these functions need further experimental evidence to enrich our knowledge.

## Conclusions

In the present study, a first comprehensive evolutionary analysis of *ABCCs* genes in *S. alfredii* Hance was carried out using a transcriptome data set. By characterizing the composition and structure of ten identified ABCC proteins, we found similar domain arrangements and conserved motifs shared by SaABCCs, indicating their similar evolutionary history. Bioinformatics analysis visualized the cluster of the putative SaABCCs and AtABCCs and composed with representative distribution of protein domains. These *SaABCC* genes exhibited tissue-specific expression profiles, particularly in roots and stems. Cd-responsive expression profiles uncovered the up-regulation of five *SaABCC* genes, among which *Sa14F190* and *Sa18F186* genes were most strongly induced. Co-expression network also suggested that *Sa14F190* gene was one of the five *SaABCCs* hub genes tensely associating with Cd stress. Heterologous expression of *Sa14F190* gene in Cd-sensitive yeast cells proved a stronger accumulation but less tolerance of Cd by *Sa14F190*-expression cell lines, hinting the possible roles of *Sa14F190* genes in Cd transport. Our findings open a new door to understand the evolution and functions of SaABCCs in *S. alfredii* Hance, unravel their roles in adapting Cd stress, provide new clues on Cd transport and detoxification in *S. alfredii* Hance, and add perspectives for studying mechanisms of PTEs tolerance and accumulation in other hyperaccumulators.

## Supplementary information

Supplementary Information 1.

Supplementary Information 2.
